# Dental and Periodontal Changes After Accelerated Correction of Lower Anterior Teeth Crowding With Periodontally Accelerated Osteogenic Orthodontics (PAOO) Procedure: A Randomized Controlled Trial

**DOI:** 10.7759/cureus.57347

**Published:** 2024-03-31

**Authors:** Hallaj I Alsino, Mohammad N. Kheshfeh, Mohammad Y Hajeer, Ahmad S Burhan, Issam Alkhouri, Heba M Al-Ibrahim, Jihad Nouman Abou Nassar

**Affiliations:** 1 Department of Orthodontics, Faculty of Dentistry, University of Damascus, Damascus, SYR; 2 Department of Oral and Maxillofacial Surgery, Faculty of Dentistry, University of Damascus, Damascus, SYR; 3 Department of Fixed Prosthodontics, Faculty of Dentistry, University of Damascus, Damascus, SYR

**Keywords:** plaque index, inter-2nd premolars width, inter-canine width, acceleration, levelling and alignment, little irregularity index, moderate crowding, lower anterior teeth crowding, periodontally accelerated osteogenic orthodontics, paoo

## Abstract

Objective

To evaluate the relative efficacy of periodontally accelerated osteogenic orthodontics (PAOO) compared to conventional fixed appliances in correcting lower anterior teeth crowding using a non-extraction treatment approach.

Material and methods

A single-center, two-arm, parallel-group randomized controlled trial was conducted on 38 patients (9 males, 29 females) with moderate crowding. These patients did not require premolar extraction and were randomly allocated into two treatment groups: the PAOO group and the conventional orthodontic treatment group. The Little Irregularity Index (LII) measured crowding intensity on pre-treatment study models. Changes in this index were recorded monthly in both treatment groups. The inter-canine width, inter-second-premolar width, plaque index (PI), gingival index (GI), and papillary bleeding index (PBI) were also measured before and after the leveling and alignment stage. Statistical analysis between the two groups was performed using Mann-Whitney U tests.

Results

For the LII, the average time for irregularity resolution was three months in the PAOO group, compared to five months in the conventional orthodontic treatment group. Regarding changes in inter-second-premolar width, the PAOO procedure led to a significant decrease in the increase of inter-second-premolar width, with an average increase of +1.52 mm compared to +2.71 mm in the control group. For the GI and PBI, it was found that their values significantly increased with PAOO application, averaging 0.18 and 0.17, respectively, compared to 0.05 and 0.07 in the control group.

Conclusions

The use of PAOO in orthodontic treatment accelerated the leveling and alignment process by 40%. Changes in the inter-canine width, the inter-second-premolar width, and the status of periodontal tissues were minimal and clinically negligible.

## Introduction

One of the frequent questions that patients usually ask at the beginning of orthodontic treatment concerns the time required to complete this treatment [1]. However, the duration of orthodontic treatment is related to many factors, such as the severity of tooth malocclusion, the technique used, the patient's compliance, and the orthodontist's skill [2]. Decreasing the duration of orthodontic treatment may help prevent unwanted effects, such as root resorption, pulpal injuries, periodontal diseases, and temporomandibular joint dysfunction [3]. This has prompted the search for techniques and measures to reduce the duration of orthodontic treatment [4].

Various methods have been used to reduce the duration of orthodontic treatment. However, it seems that surgical procedures, applied more professionally, might yield outcomes that are more clearly evident when the orthodontic treatment duration is short [5]. This is due to the regional acceleratory phenomenon (RAP) that occurs as a result of surgical intervention, leading to an acceleration of orthodontic tooth movement [6]. Several RAP-based surgical techniques have been employed to speed up tooth movements, including corticision [7], conventional corticotomy [8,9], piezocision-based flapless corticotomy [10-12], and laser-assisted flapless corticotomy [13].

Wilcko WM et al. also emphasized the concept of RAP in accelerating tooth movement and published the first case report in 2001 under the name periodontally accelerated osteogenic orthodontics (PAOO) [6]. Subsequently, several studies have been published on the effectiveness of PAOO in accelerating tooth movement, increasing alveolar bone thickness, and reducing the risk of root resorption [6,14-19].

Reviewing the available literature reveals a scarcity of clinical studies evaluating dentoalveolar changes following the PAOO procedure. Only one clinical trial evaluated Little's Irregularity Index (LII) and dental arch width [20]. In their clinical trial, which included 30 patients with Class I with severe crowding, LII, inter-canine width, and inter-second-premolar width were evaluated in the PAOO group compared to the conventional orthodontic group. However, in this study, there were two different treatment techniques in each group, as the plan in the control group included the extraction of the first premolars during the course of treatment. This was an obvious confounding factor in the interpretation of the results.

The current study aimed to evaluate the effectiveness of combining the PAOO procedure with non-extraction orthodontic treatment for patients with Class I malocclusion and moderate crowding, in comparison with a control group receiving ordinary orthodontic treatment, in terms of LII, dental arch width, and periodontal status.

## Materials and methods

Settings and study design

This study was conducted as a two-arm, parallel-group randomized controlled trial at the Department of Orthodontics, Faculty of Dentistry, University of Damascus, between January 1, 2021, and February 1, 2022. ClinicalTrials.gov has filed this study under the number NCT05390320. The project was funded by the University of Damascus Dental School Postgraduate Research Budget (Ref no: 501100020595). The University of Damascus Local Research Ethics Committee approved this study (UDDS-510-08022020/SRC-2110).

Estimation of the sample size

Using an alpha level of 0.05 and a confidence interval of 95%, the sample size was determined using the Minitab® 19.1 program (Minitab LLC, Pennsylvania, USA). The focus factor in the current study was the change in LII. According to Uribe F et al., the SD for this variable was 1.57 mm [21]. The least clinically significant difference to be detected was a change of 2 mm in the Little's Irregularity Index. Using an independent-samples t-test, the required sample size was 18 patients for each group. However, with an assumed withdrawal rate of 5%, the required sample size was increased to 19 patients for each group.

Patients' recruitment and inclusion in the study

The inclusion criteria were as follows: (1) Normal vertical growth pattern; (2) skeletal class I malocclusion (ANB=2-4); and (3) moderate crowding of lower anterior teeth (i.e., 4-6 millimeters of tooth size-arch length discrepancy), where the treatment plan did not require extraction of any dental units; (4) absence of anterior or posterior crossbites; (5) 1-4 mm of overbite and 1-3 mm of overjet; (6) adult healthy patients of both sexes within an age range of 18-28 years; (7) optimal dental health. The exclusion criteria were as follows: (1) medical, social, and psychological reasons for not undergoing oral surgery; (2) periodontal disorders; (3) inadequate dental care; (4) history of orthodontic therapy.

**Figure 1:**
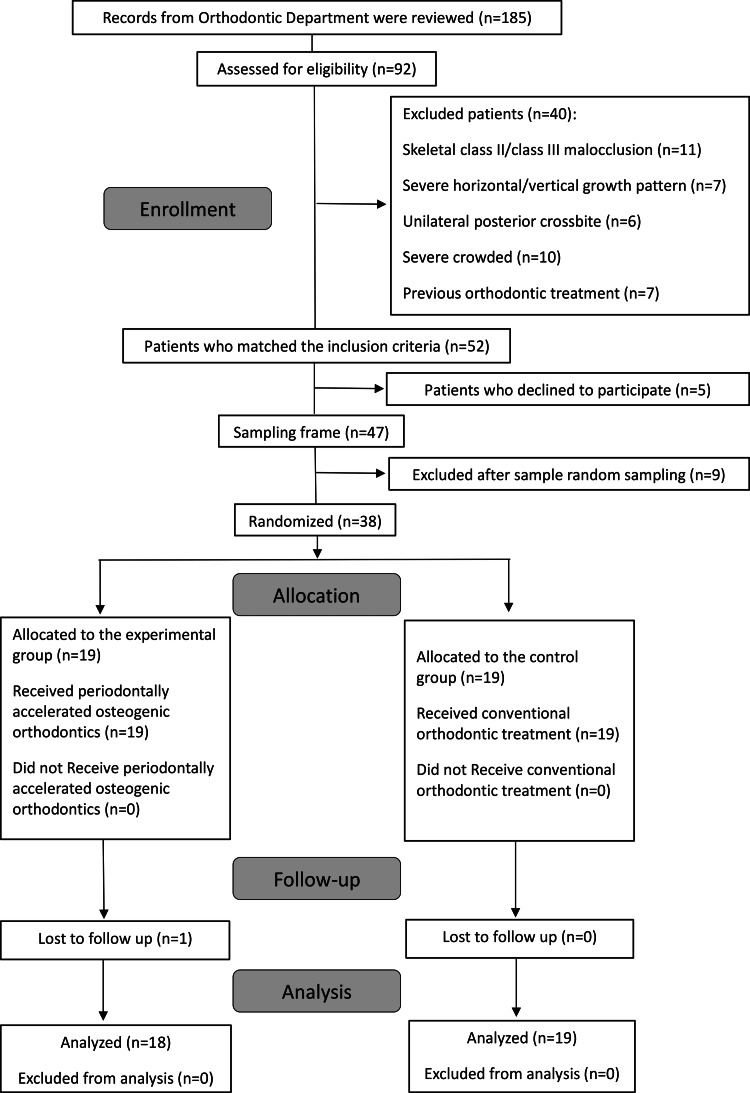
The flow diagram of patient enrollment, allocation, follow-up, and entry into data analysis.

At the Department of Orthodontics, University of Damascus, 92 patients were examined, and 52 patients who fulfilled the inclusion criteria were identified. When the research project was presented to the patients, 47 agreed to participate. Consequently, 38 patients (9 males, 29 females) were randomly selected, as shown in the CONSORT flow diagram (Figure 1). Patients received information sheets, and informed consent forms were collected after obtaining permission.

Randomization, allocation concealment, and blinding

A 1:1 allocation ratio list of random numbers generated by Minitab® Version 19.1 (Minitab Inc., Pennsylvania, USA) was used to divide the patients between the two groups. Thirty-eight patients were randomly assigned to one of two groups: the PAOO group or the conventional orthodontic treatment group without PAOO. The allocation sequence was concealed using a series of opaque, sealed envelopes. It was not feasible to blind the patients or the doctors; therefore, blinding was applied only during the data analysis phase. An academic member not involved in the study executed the randomization process and assigned the participants to the two groups.

Surgical intervention in the experimental group

**Figure 2:**
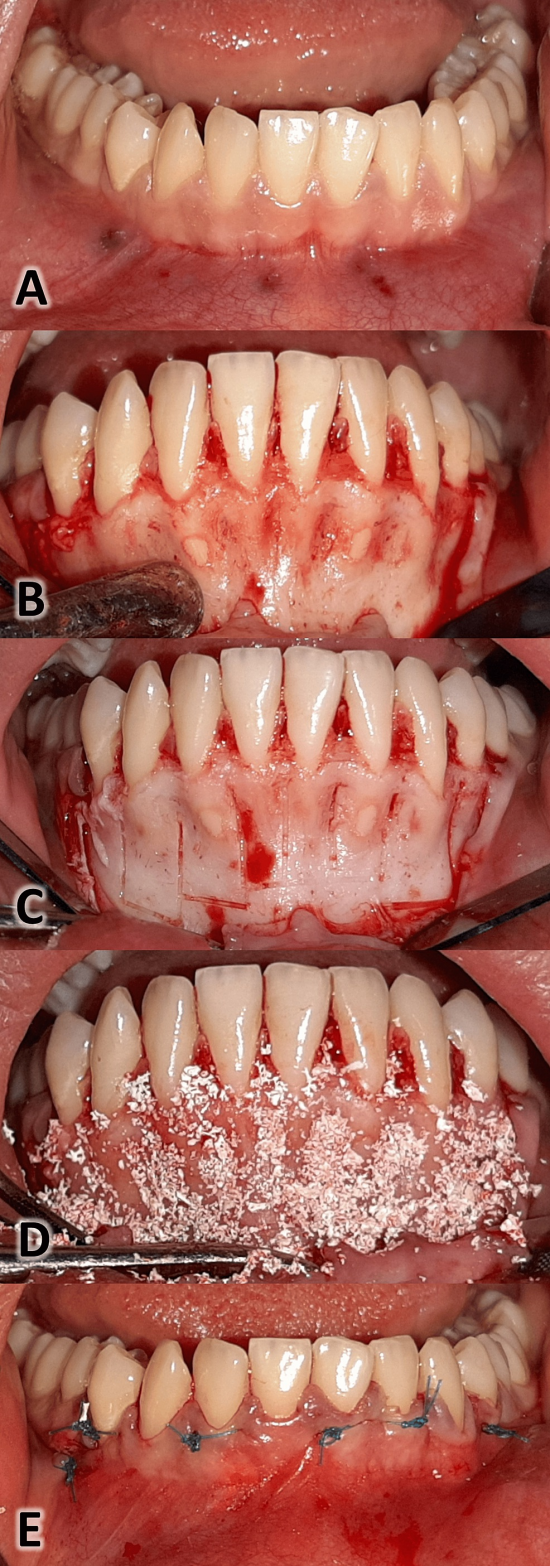
The surgical procedure in the experimental group. A: Local anesthetic (2% lidocaine HCL-1:80000 epinephrine). B: A full-thickness labial flap was reflected. C: Using piezosurgery, selective cortical cuts were performed. D: The xenograft was placed in its position. Its particle size ranged from 0.2 mm to 1.0 mm (Bone-D®, MedPark Co., Busan, Korea). E: Two-metric nylon 3/0 sutures were used on a reverse cutter needle (Shandong, China) to replace the labial flap.

All surgical interventions associated with the PAOO procedure were performed under local anesthesia at the Department of Oral and Maxillofacial Surgery, Faculty of Dentistry, Damascus University, by the principal author (Alsino HI). One of the co-authors (Alkhouri I) carried out the surgical procedures according to Bahammam MA [22] (Figure 2). From the distal surface of the lower right canine to the distal surface of the lower left canine, a full-thickness gingival flap was elevated buccally. Then, selective alveolar bone cortical cuts were made using a piezo-surgical device, following the vertical cut lines below the crestal bone between the roots of the lower anterior teeth. These vertical cut lines connected to a horizontal line 2-3 mm below the apex of the roots of the lower anterior teeth. After inserting the xenograft (Bone-D®, MedPark Co., Busan, Korea), with particle diameters ranging between 0.2 and 1.0 mm, the incision was sutured. Amoxicillin 500 mg was recommended three times a day for a week post-surgery. If needed, patients were instructed to take 500 milligrams of paracetamol.

Orthodontic procedures for both treatment groups

Participants at the Department of Orthodontics, Faculty of Dentistry, Damascus University, underwent treatment with a fixed orthodontic appliance, administered by the principal researcher (Alsino HI). The PAOO group initially received surgical intervention, followed by the application of an MBT 0.022-inch bracket slot (Votion™, Ortho Technology®, Florida, USA) prescription on the 7th day after the surgery. Immediately after applying the fixed orthodontic appliance, the first archwire for alignment, a 0.012-inch Nickel-Titanium (NiTi) archwire, was placed in both treatment groups.

Patients in the PAOO group were scheduled for regular visits every two weeks, and every three weeks in the conventional orthodontic group, for the application of archwire sequences until the end of the leveling and alignment phase, i.e., until the application of a 0.019 x 0.025-inch stainless steel archwire. The archwires were replaced when the new archwire did not cause excessive pressure on crowded teeth [11].

Outcome measures

First: Dentoalveolar Changes

Little's irregularity index (LII):** **The LII was measured on mandibular study models at the following assessment times: before the treatment intervention (T0), one month after the insertion of the first orthodontic archwire (T1), two months after (T2), three months after (T3), four months after (T4), and at the end of the alignment stage (T5), which was determined when the LII was less than 1 mm.

**Figure 3:**
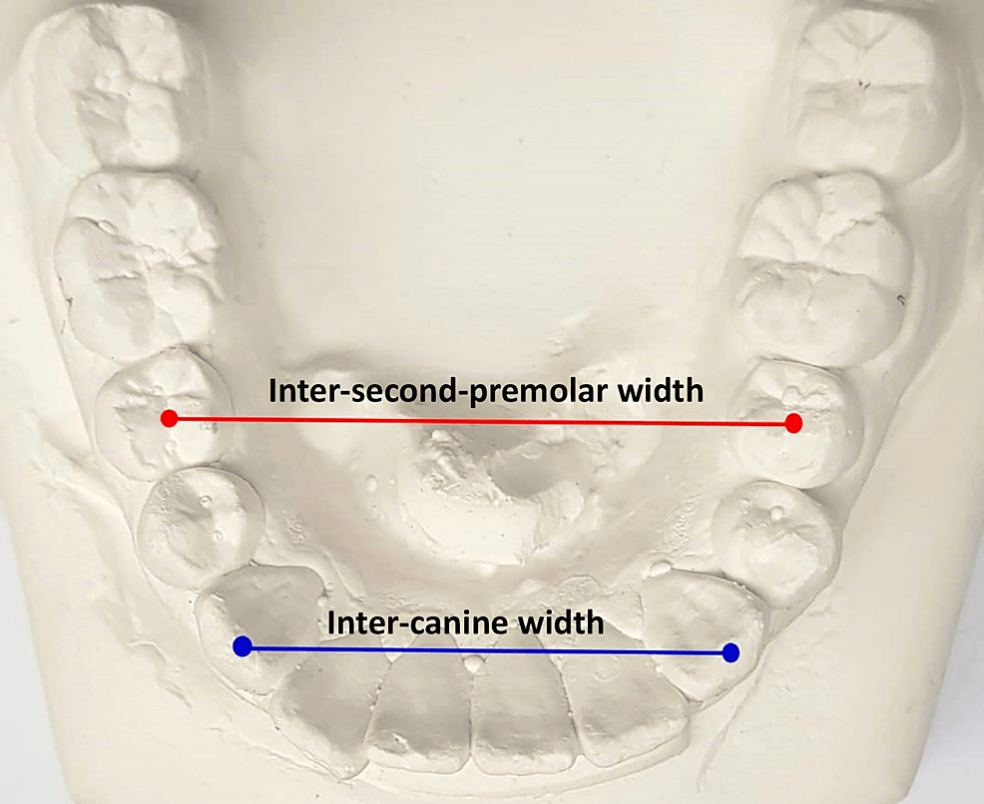
Inter-canine and inter-second-premolar widths measured on study models.

Inter-canine width:** **The distance between the crown tips of the right and left canines was measured on mandibular dental models before the start of the orthodontic procedure (T0) and following the completion of the leveling and alignment stage (T1) (Figure 3).

Inter-second-premolar width: The distance between the central fossae of the second premolars was measured on mandibular dental models before the beginning of orthodontic treatment (T0) and at the end of the leveling and alignment stage (T1).

Second: Periodontal Condition Changes

Periodontal parameters were measured before the beginning of treatment (T0) and at the end of the leveling and alignment stage (T1) in both groups. The measured periodontal parameters included the plaque index (PI), gingival index (GI), and papillary bleeding index (PBI). After drying the tooth surface, the plaque index was evaluated by moving the gingival probe over the gingival margin of the following tooth surfaces: buccal, lingual, mesiobuccal, and distobuccal [23-24]. The PI value for each patient was determined by averaging the values of the six lower anterior teeth. The recorded values were categorized according to the following scores: (0) no plaque, (1) a layer of plaque attached to the free gingival border and the tooth's surrounding tissue, visible only after applying a disclosing solution or passing the gingival probe over the tooth's surface; (2) moderate accumulation of soft deposits within the gingival pocket or on the tooth and gingival margin, visible to the naked eye; and (3) severe accumulation of plaque in the interdental spaces and along the gingival margin, visible to the naked eye.

The gingival index was evaluated using William’s probe to assess the severity of gingivitis at the buccal gingival margin, lingual gingival margin, mesiobuccal papilla, and distobuccal papilla [23]. The GI value for each patient was determined by averaging the values of the six lower anterior teeth. The recorded values were categorized according to the following scores: (0) no inflammation, (1) mild inflammation: slight color change and bleeding on probing; (2) moderate inflammation: redness, edema, hypertrophy, and bleeding on pressure; and (3) severe inflammation: marked redness, hypertrophy, tendency to spontaneous bleeding, and ulceration.

The papillary bleeding index was evaluated using William’s probe to assess the gingival health through the natural susceptibility of the gingival papillae to bleeding. A periodontal probe is placed into the gingival sulcus at the base of the papilla on the mesial side, then pushed coronally to the papilla's end, and repeated on the distal aspect of the papilla. The recorded levels of bleeding severity are: (0) no bleeding, (1) a single, separate bleeding point, (2) multiple, separated bleeding points or a single line of blood; (3) the interdental triangle covers with blood immediately following probing; and (4) profuse bleeding following probing, with blood flowing straight into the marginal sulcus.

Statistical analysis

Anderson-Darling Normality tests using Minitab® Version 19.1 revealed an abnormal distribution of the data, necessitating the use of non-parametric tests. Friedman's test was used to detect significant changes in LII over time, and Wilcoxon signed-rank tests were employed for pairwise comparisons within the same group. Additionally, the Mann-Whitney U test identified significant variations between the two groups. Regarding inter-canine width, inter-second-premolar width, and periodontal condition changes, Wilcoxon signed-rank tests identified significant variations among participants within the same group, and Mann-Whitney U tests detected significant changes between the two groups.

Version 24.0 of SPSS® Statistics (IBM Corp., Armonk, NY, USA) was used for all statistical analyses. The normality of distributions was tested using the Anderson-Darling test. The significance of gender distribution disparities was assessed using the Chi-square test. The Friedman test was used to determine the significance of the changes for each group separately in the Little Irregularity Index over time. Mann-Whitney test was used to determine the significance of changes between the two groups at different evaluation times. Wilcoxon signed-ranks test was used separately for pairwise comparisons of the changes in the irregularity index value over time within each group. The results were regarded as significant when the p-value was below 0.05. Furthermore, Bonferroni's correction was employed when multiple comparisons were found.

## Results

Thirty-seven patients (28 females and nine males) were included in the current trial. One patient withdrew from the trial, and another was excluded from the analyses. The PAOO group comprised 18 patients (14 females, four males, with an average age of 20.83±3.53), while the traditional orthodontic treatment group included 19 patients (14 females and five males, with an average age of 21.94±2.01). Basic sample characteristics are provided in Table 1.

**Table 1:** Baseline sample characteristics. PAOO: Periodontally accelerated osteogenic orthodontics; Trad: traditional orthodontic treatment. ^†^ Chi-Square; ^‡^ Independent-samples t test; NS: non-significant at P>0.05.

Variable		PAOO (18 patients)	Trad (19 patients)	P-value	Significance
Gender	Male	4	5	0.772^†^	NS
	Female	14	14		
Age (in years): mean ± SD		20.83 (3.53)	21.94 (2.01)	0.074^‡^	NS
Crowding (in mm): mean + SD		4.94 (0.78)	4.89 (0.80)	0.850^‡^	NS

As measured by Little's index, the total time needed to achieve a lack of irregularity was only three months in the PAOO group, compared to five months in the control group. The changes observed in the LII were quicker in the PAOO group over the observation period (Table 2). In the PAOO group, the severity of irregularity showed a significant clinical and statistical decrease after one month of treatment (a mean of -4.31 mm) compared to the control group (a mean of -2.24 mm). Pairwise comparisons between different assessment times are provided in Table 3 for each group. The difference in lower anterior teeth irregularity between the two groups is illustrated in Table 4. After four and five months of treatment, there were no significant differences between the two groups in terms of the severity of crowding, as both groups achieved complete leveling and alignment of the lower anterior teeth.

**Table 2:** Descriptive and inferential statistics of Little's Irregularity Index changes over time in each group. ^† ^Friedman's test, * significant at P<0.001, PAOO: Periodontally Accelerated Osteogenic Orthodontics; Trad: Traditional orthodontics treatment; SD: Standard deviation; Min: Minimum; Max: Maximum; T0: before starting treatment; T1: one month following the beginning of treatment; T2: after two months; T3: after three months; T4: after four months; and T5: at the end of the leveling and alignment phase.

Little's Irregularity Index	Time	PAOO (n=18)					Trad (n=19)				
		Mean	SD	Min	Max	P-value^†^	Mean	SD	Min	Max	P-value^†^
	T0	4.94	0.78	4.00	6.00	<0.001*	4.89	0.80	4.00	6.00	<0.001*
	T1	0.63	0.57	0.00	2.50		2.65	1.01	1.00	4.50	
	T2	0.03	0.12	0.00	0.50		1.39	0.76	0.25	2.50	
	T3	0.01	0.02	0.00	0.10		0.40	0.50	0.00	1.50	
	T4	0.00	0.02	0.00	0.12		0.06	0.23	0.00	1.00	
	T5	0.00	0.02	0.00	0.10		0.01	0.05	0.00	0.25	

**Table 3:** Results of the post-hoc significance testing for pairwise comparisons between assessment times in each group. ^†^ The Wilcoxon signed-ranks tests were employed for pairwise comparisons, with the Bonferroni adjustment of alpha level (i.e., 0.05/15=0.003), *: P<0.003 (significant difference). PAOO: Periodontally Accelerated Osteogenic Orthodontics; Trad: traditional orthodontic treatment; T0: before starting treatment; T1: one month following the beginning of treatment; T2: after two months; T3: after three months; T4: after four months; and T5: at the end of the leveling and alignment phase.

Time points	PAOO (n=18)	Trad (n=19)
	P-value^†^	P-value^†^
T0-T1	<0.001*	<0.001*
T0-T2	<0.001*	<0.001*
T0-T3	<0.001*	<0.001*
T0-T4	<0.001*	<0.001*
T0-T5	<0.001*	<0.001*
T1-T2	0.001*	<0.001*
T1-T3	0.001*	<0.001*
T1-T4	0.001*	<0.001*
T1-T5	0.001*	<0.001*
T2-T3	0.180	<0.001*
T2-T4	0.285	<0.001*
T2-T5	0.285	<0.001*
T3-T4	0.655	0.004
T3-T5	1.000	0.004
T4-T5	0.180	0.180

**Table 4:** Descriptive and inferential statistics of Little's Irregularity Index changes and the comparisons between the two groups at each assessment time. ^† ^Mann-Whitney U Test, with the Bonferroni adjustment of alpha level (i.e., 0.05/6=0.008), *: P<0.008 (significant difference). PAOO: Periodontally Accelerated Osteogenic Orthodontics; Trad: Traditional orthodontics treatment; Min: Minimum; Max: Maximum; T0: before starting treatment; T1: one month following the beginning of treatment; T2: after two months; T3: after three months; T4: after four months; and T5: at the end of the leveling and alignment phase.

Little's Irregularity Index	PAOO (n=18)				Trad (n=19)				P-value^†^
	Mean	SD	Min	Max	Mean	SD	Min	Max	
T0	4.94	0.78	4.00	6.00	4.89	0.80	4.00	6.00	0.877
T1	0.63	0.57	0.00	2.50	2.65	1.01	1.00	4.50	<0.001*
T2	0.03	0.12	0.00	0.50	1.39	0.76	0.25	2.50	<0.001*
T3	0.01	0.02	0.00	0.10	0.40	0.50	0.00	1.50	0.001*
T4	0.00	0.02	0.00	0.12	0.06	0.23	0.00	1.00	0.955
T5	0.00	0.02	0.00	0.10	0.01	0.05	0.00	0.25	0.585

The increase in inter-canine width was more pronounced, although not statistically significant, in the PAOO group compared to the control group (a mean of +0.77 mm versus +0.36 mm, respectively). However, the inter-second-premolar width significantly increased in the control group compared to the PAOO group (a mean of +2.71 mm versus +1.52 mm, respectively), as shown in Table 5.

**Table 5:** Descriptive and inferential statistics of the changes in the inter-canine width and inter-second-premolar width for the two groups. ^†^Mann-Whitney U Test, *: P<0.05 (significant difference). PAOO: Periodontally Accelerated Osteogenic Orthodontics; Trad: Traditional orthodontics treatment; ICW: Inter-canine width; IPW: Inter-second-premolar width; Min: Minimum; Max: Maximum; T0: Before starting treatment; T1: End of leveling and alignment phase.

T0-T1	PAOO (n=18)				Trad (n=19)				P-value^†^
	Mean	SD	Min	Max	Mean	SD	Min	Max	
ICW	0.77	0.52	0.00	2.50	0.36	0.83	-1.50	1.50	0.265
IPW	1.52	1.11	0.25	5.00	2.71	1.09	0.50	5.00	0.001*

The study's findings indicated a statistically significant increase in the gingival and papillary bleeding indexes in the PAOO group, with a mean increase of 0.18 and 0.17, respectively. In contrast, the traditional orthodontic treatment group experienced only a mean increase of 0.05 and 0.07, respectively. Regarding the changes in the plaque index, the two groups did not differ statistically significantly (P = 0.988, Table 6).

**Table 6:** Descriptive and inferential statistics of changes to the periodontal indexes used in the current study in both groups. †Mann-Whitney U Test, *: P<0.05 (significant difference). PAOO: Periodontally Accelerated Osteogenic Orthodontics; Trad: Traditional orthodontics treatment; PI: Plaque Index; GI: Gingival Index; PBI: Papillary Bleeding Index; Min: Minimum; Max: Maximum; T0: Before starting treatment; T1: End of leveling and alignment phase.

T0-T1	PAOO (n=18)				Trad (n=19)				P-value^†^
	Mean	SD	Min	Max	Mean	SD	Min	Max	
GI	0.18	0.17	0.00	0.75	0.05	0.10	-0.13	0.25	0.019*
PI	0.01	0.25	-0.54	0.46	0.37	0.18	-0.25	0.38	0.988
PBI	0.17	0.14	0.04	0.63	0.07	0.15	-0.21	0.33	0.047*

Harms

One postsurgical complication was observed in a patient from the experimental group. Within ten days after the surgery, the expected healing did not occur, and swelling and redness appeared in the mucous membrane, leading to the extrusion of the bone graft through the mucous membrane. The patient received daily wound cleaning and antibiotic treatment, resulting in mild gingival recession observed one month postoperatively.

## Discussion

This is the first parallel-group randomized controlled clinical trial to evaluate the effectiveness of the PAOO procedure in correcting moderate crowding without extraction, compared to a control group receiving traditional treatment. The evaluation included the Little Index of Irregularity, intercanine and inter-second-premolar widths, and the status of the periodontal tissues following treatment. The patients participating in the study ranged in age from 18 to 28, with a mean age of 21.40 ± 2.87. This study included only adult patients to eliminate the potential impact of growth factors and metabolic changes on dental movement in young individuals [2], aligning with several previous papers that selected adult patients for acceleratory interventions in orthodontic treatment [19,22].

Changes in LII

Reviewing the available literature revealed the absence of any study that evaluated the impact of the PAOO on the alignment of the crowded anterior teeth compared with a group of patients treated without a surgical intervention using Little's index of irregularity as an assessment method.

The mean value of Little's index decreased significantly after one month at T1 in the experimental group (i.e., -4.31 mm), compared to the decrease in the mean value of Little's index after one month at T1 in the conventional group (i.e., -2.24 mm). This difference between the two treatment groups was statistically and clinically significant (P<0.001), possibly due to the surgical intervention triggering the RAP, which leads to an accelerated rate of tooth movement for 1-2 months [6,8].

Our trial's results were consistent with Gibreal O et al.'s trial, where flapless corticotomy was used in the experimental group, alongside the extraction of the first premolar (performed in both groups), showing significantly better results [11]. This contrasts with Uribe F et al. [21], whose study included an experimental group that received flapless corticotomy and the treatment regimen of self-ligating brackets applied to both groups, highlighting the influence of additional accelerators, like self-ligating brackets, present in both groups.

The inter-canine and inter-second-premolar widths

The slight increase in inter-canine width in the orthodontic group with PAOO compared to the traditional orthodontic treatment group (a mean of +0.41 mm) was not statistically significant (P>0.05). These results agreed with those in the study of Al-Naoum F et al. [20]. The change in inter-second-premolar width in the traditional orthodontic treatment group increased significantly, P<0.01, but may not be considered clinically significant compared to the amount of change in the orthodontic group with PAOO. Thus, these results differed from the study of Al-Naoum F et al., where they concluded that the inter-second-premolar width increased significantly in the orthodontic group with PAOO compared to the control group. One possible explanation for this difference could be that the two samples in the Al-Naoum F study were not comparable because, in the traditional orthodontic treatment group, treatment was performed with the extraction of the first premolars, while in the current trial, both groups were treated using a non-extraction approach.

The condition of periodontal tissues

The RAP explains the high average changes in the values of the GI and the bleeding index upon probing in the orthodontic group with PAOO. Thus, these results are consistent with those of Aboul-Ela SM et al.'s study, which found that the RAP requires a long period for complete healing, which may cause an increase in the value of the gingivitis index [25].

Limitations

In this study, the surgical intervention was only applied to the lower dental arch, so the effectiveness of this procedure on upper dental crowding was not evaluated. Additionally, this study did not examine patient-centered outcomes, skeletal outcomes, collateral harms (such as root resorption and scarring), tooth vitality, or the role of gender in resolving crowding.

Generalizability

This clinical trial was conducted according to strict inclusion criteria, including a specific type of malocclusion and lower middle crowding, with a sample aged 18 to 28, the findings of this trial can only be generalized to patients with similar characteristics to those included in the study.

## Conclusions

PAOO is a highly effective procedure for quickly correcting irregularities in tooth alignment, with an acceleration rate of 40% compared to the control group. By applying the PAOO procedure in orthodontic treatment, no clinically important changes were observed between the two groups regarding arch width measured between the canines and second premolars. The changes observed in the periodontal tissues in the PAOO group were minimal and had little impact from a clinical standpoint. Therefore, using PAOO did not have any noticeable negative effects on the periodontal tissues.
